# Elevated protein concentrations in newborn blood and the risks of autism spectrum disorder, and of social impairment, at age 10 years among infants born before the 28th week of gestation

**DOI:** 10.1038/s41398-018-0156-0

**Published:** 2018-06-08

**Authors:** Steven J. Korzeniewski, Elizabeth N. Allred, T. Michael O’Shea, Alan Leviton, Karl C. K. Kuban, for the ELGAN study investigators, Kathleen Lee, Anne McGovern, Jill Gambardella, Susan Ursprung, Ruth Blomquist Kristen Ecklund, Haim Bassan, Samantha Butler, Adré Duplessis, Cecil Hahn, Catherine Limperopoulos, Omar Khwaja, Janet S. Soul, Bhavesh Shah, Karen Christianson, Frederick Hampf, Herbert Gilmore, Susan McQuiston, Camilia R. Martin, Colleen Hallisey, Caitlin Hurley, Miren Creixell, Jane Share, Linda J. Van Marter, Sara Durfee, Robert M. Insoft, Jennifer G. Wilson, Maureen Pimental, Sjirk J. Westra, Kalpathy Krishnamoorthy, Cynthia Cole, John M. Fiascone, Janet Madden, Ellen Nylen, Anne Furey Roy McCauley, Paige T. Church, Cecelia Keller, Karen J. Miller, Francis Bednarek, Mary Naples, Beth Powers, Jacqueline Wellman, Robin Adair, Richard Bream, Alice Miller, Albert Scheiner, Christy Stine, Richard Ehrenkranz, Joanne Williams, Elaine Romano, Cindy Miller, Nancy Close, Elaine Romano, Joanne Williams, T. Michael O’Shea, Debbie Gordon, Teresa Harold, Barbara Specter, Deborah Allred, Robert Dillard, Don Goldstein, Deborah Hiatt, Gail Hounshell, Ellen Waldrep, Lisa Washburn, Cherrie D. Welch, Stephen C. Engelke, Sherry Moseley, Linda Pare, Donna Smart, Joan Wilson, Ira Adler, Sharon Buckwald, Rebecca Helms, Kathyrn Kerkering, Scott S. MacGilvray, Peter Resnik, Carl Bose, Gennie Bose, Lynn A. Fordham, Lisa Bostic, Diane Marshall, Kristi Milowic, Janice Wereszczak, Mariel Poortenga, Dinah Sutton, Bradford W. Betz, Steven L. Bezinque, Joseph Junewick, Wendy Burdo-Hartman, Lynn Fagerman, Kim Lohr, Steve Pastyrnak, Dinah Sutton, Carolyn Solomon, Ellen Cavenagh, Victoria J. Caine, Nicholas Olomu, Joan Price, Nigel Paneth, Padmani Karna, Madeleine Lenski, Michael D. Schreiber, Grace Yoon, Kate Feinstein, Leslie Caldarelli, Sunila E. O’Connor, Michael Msall, Susan Plesha-Troyke, Daniel Batton, Beth Kring, Karen Brooklier, Beth Kring, Melisa J. Oca, Katherine M. Solomon

**Affiliations:** 1 Department of Obstetrics and Gynecology, Wayne State University School of Medicine, Detroit, MI USA; 2 Departments of Neurology, Boston Children’s Hospital, and Harvard Medical School, Boston, MA USA; 3 Department of Pediatrics, University of North Carolina, Chapel Hill, NC USA; 4 Departments of Pediatrics, Boston Medical Center and Boston University, Boston, MA USA; 5 Children’s Hospital, Boston, MA USA; 6 Baystate Medical Center, Springfield, MA USA; 7 Beth Israel Deaconess Medical Center, Boston, MA USA; 8 Brigham and Women’s Hospital, Boston, MA USA; 9 Massachusetts General Hospital, Boston, MA USA; 10 Floating Hospital for Children at Tufts Medical Center, Boston, MA USA; 11 UMass Memorial Health Care, Worcester, MA USA; 12 Yale University School of Medicine, New Haven, CT USA; 13 Wake Forest University Baptist Medical Center and Forsyth Medical Center, Winston-Salem, NC USA; 14 University Health Systems of Eastern Carolina, Greenville, NC USA; 15 North Carolina Children’s Hospital, Chapel Hill, NC USA; 16 Helen DeVos Children’s Hospital, Grand Rapids, MI USA; 17 Sparrow Hospital, Lansing, MI USA; 18 Michigan State University, East Lansing, MI USA; 19 University of Chicago Medical Center, Chicago, IL USA; 20 William Beaumont Hospital, Royal Oak, MI USA

## Abstract

Among the 1 of 10 children who are born preterm annually in the United States, 6% are born before the third trimester. Among children who survive birth before the 28th week of gestation, the risks of autism spectrum disorder (ASD) and non-autistic social impairment are severalfold higher than in the general population. We examined the relationship between top quartile inflammation-related protein concentrations among children born extremely preterm and ASD or, separately, a high score on the Social Responsiveness Scale (SRS total score ≥65) among those who did not meet ASD criteria, using information only from the subset of children whose DAS-II verbal or non-verbal IQ was ≥70, who were assessed for ASD, and who had proteins measured in blood collected on ≥2 days (*N* = 763). ASD (*N* = 36) assessed at age 10 years is associated with recurrent top quartile concentrations of inflammation-related proteins during the first post-natal month (e.g., SAA odds ratio (OR); 95% confidence interval (CI): 2.5; 1.2–5.3) and IL-6 (OR; 95% CI: 2.6; 1.03–6.4)). Top quartile concentrations of neurotrophic proteins appear to moderate the increased risk of ASD associated with repeated top quartile concentrations of inflammation-related proteins. High (top quartile) concentrations of SAA are associated with elevated risk of ASD (2.8; 1.2–6.7) when Ang-1 concentrations are below the top quartile, but not when Ang-1 concentrations are high (1.3; 0.3–5.8). Similarly, high concentrations of TNF-α are associated with heightened risk of SRS-defined social impairment (*N* = 130) (2.0; 1.1–3.8) when ANG-1 concentrations are not high, but not when ANG-1 concentrations are elevated (0.5; 0.1–4.2).

## Introduction

Children born very preterm are at increased risks of autism spectrum disorder (ASD)^1,2^ as well as a range of social limitations that do not meet ASD diagnostic criteria^3,4^. In the ELGAN study of children born prior to the 28th week of gestation, we used the Social Responsiveness Scale (SRS)^5^ to assess the constellation of problems referred to as the “preterm behavioral phenotype”^6^ among children who did not meet rigorous ASD criteria at age 10 years and found that the prevalence was fourfold greater than was expected based on general population norms^7^.

Neonatal systemic inflammation (and related phenomena^8^) appear to raise the risk of developing brain alterations^9^. By increasing the risk of structural brain abnormalities, neonatal systemic inflammation might also contribute to an increased risk of ASD^10–12^ and of social limitations assessed by the SRS^13–15^ among children who are not considered autistic. Growth factors with neurotrophic properties have the potential to minimize this risk^16–18^.

We wanted to compare the systemic inflammation and neurotrophic-protein newborn blood profiles among children who met rigorously-defined ASD criteria, and among children who did not meet these criteria, but who nevertheless had very high SRS scores at age 10 years, with the profiles of children who neither met ASD criteria nor had very high SRS scores. By posing this question, we in-effect sought evidence that supported or refuted the hypothesis that the two entities are characterized by different blood protein profiles during the first post-natal month.

## Methods

The ELGAN study, a multi-center prospective, observational study of the risk of structural and functional neurologic disorders in infants born before the 28th week of gestation^19^, enrolled 1506 infants born before the 28th week. Of these, 1198 children survived to 10 years; 889 (92%) of the 966 children who were actively recruited for follow-up based on the availability of blood samples collected during their first post-natal month provided informed consent to participate at age 10 years. Enrollment and consent procedures used for all patients in this follow-up study were approved by the institutional review boards of all participating institutions. The sample size was derived to provide power to perceive a doubling or halving of risk. Additional details about the study design^19^, and about the assessment procedures used at age 10 years^1,20^, are provided in prior publications.

### Newborn variables

The gestational age estimates were based on a hierarchy of the quality of available information. Most desirable were estimates based on the dates of embryo retrieval or intrauterine insemination or fetal ultrasound before the 14th week (62%). When these were not available, reliance was placed on a ≥14 weeks’ fetal ultrasound (29%), last menstrual period (LMP) without fetal ultrasound (7%), and gestational age recorded in the log of the neonatal intensive care unit (NICU) (1%). The birthweight *Z*-score is the number of standard deviations the infant’s birthweight is above or below the median weight of infants at the same gestational age in a standard dataset^21^.

### Procedures for the assessments at age 10 years

Families willing to participate were scheduled for one visit during which all of the measures reported here were administered in 3–4 h, including breaks. The assessments were selected to provide the most comprehensive information about neurocognitive and academic function in one testing session. While the child was tested, the parent or caregiver completed questionnaires regarding the child’s medical and neurological status and behavior.

### General cognitive ability

General cognitive ability (or intelligent quotient (IQ)) was assessed with the School-Age Differential Ability Scales–II (DAS-II) Verbal and Non-verbal Reasoning scales^22^. We classified children into two groups based on verbal or non-verbal components ≥70 (yes vs. no).

### ASD assessment

The evaluation of ASD characteristics was conducted sequentially with three instruments and is described in detail elsewhere^20^. Briefly, children who had a Social Communication Questionnaire (SCQ)^23^ score ≥11 were brought back on another day to be evaluated with the Autism Diagnostic Interview-Revised (ADI-R)^24^. Those who satisfied modified criteria^20^ were assessed with the Autism Diagnostic Observation Schedule, Second Version (ADOS-2)^25^, which served as the criterion measure of ASD in this study. Eleven children were assessed by ADOS-2 who were not assessed by the ADI-R; eight children were tested based on a prior clinical diagnosis of ASD and/or having symptoms of ASD during cognitive testing according to the site psychologist, and the parents of two children did not complete the ADI-R assessment.

### Indicator of social dysfunction: Social Responsiveness Scale ≥65

The SRS identifies social impairment and quantifies its severity^5^. This 65-item instrument provides a total score reflecting severity of social deficits, as well as five subscale scores: social awareness, social cognition, social communication, social motivation, and restricted interests and repetitive behavior. We dichotomized scores at 65 (i.e., the 96th percentile in the general population), as have others^26^, which is higher than the published threshold of 60 (i.e., the 84th percentile) used to indicate moderate deficiencies in reciprocal soci°al behavior that are “clinically significant” in the general population^5^.

### Blood spot collection and measurement

Drops of blood were collected on filter paper on the first post-natal day (range: 1–3 days), the 7th post-natal day (range: 5–8 days), the 14th post-natal day (range: 12–15 days), the 21st post-natal day (range: 19–23 days), and the 28th post-natal day (range: 26–29). All blood was from the remainder of specimens obtained for clinical indications. Dried blood spots were stored at −70 °C in sealed bags with a desiccant until processed. Details about the elution of proteins from the blood spots are provided elsewhere^27^. The total protein concentration in each eluted sample was determined by BCA assay (Thermo Scientific, Rockford, IL) using a multi-label Victor 2 counter (Perkin Elmer, Boston, MA) and the measurements of each analyte normalized to mg total protein.

### Proteins measured

The Genital Tract Biology Laboratory at the Brigham and Women’s Hospital in Boston Massachusetts eluted all blood spots as previously described and measured all proteins reported here. The laboratory used the Meso Scale Discovery to measure: C-reactive protein, serum amyloid A (SAA), myloeperoxidase, interleukin-1 β (IL-1β), IL-6, IL-6 receptor (IL-6R), tumor necrosis factor-α (TNF-α), TNF receptor-1 (TNFR-1), TNFR-2, IL-8 (CXCL8), regulated upon activation, normal T-cell expressed and secreted (RANTES; CCL5), intercellular adhesion molecule -1 (ICAM-1; CD54), vascular cell adhesion molecule-1 (VCAM-1; CD106), vascular endothelial growth factor (VEGF), VEGF receptor-1 (VEGFR-1, also known as sFLT-1), VEGFR-2 (KDR), insulin-like growth factor-1 (IGF) binding protein-1 (IGFBP-1), thyroid-stimulating hormone, metalloproteinase (MMP)-9, and erythropoietin (EPO).

A multiplex immunobead assay manufactured by R&D Systems (Minneapolis, MN) and a MAGPIX Luminex reader (R&D Systems) were used to measure angiopoietin-1 (Ang-1), Ang-2, placenta growth factor (PIGF), neurotrophin-4 (NT-4), brain-derived neurotrophic factor (BDNF), and basic fibroblastic growth factor (bFGF). ELISA (R&D Systems) was used to measure IGF-1.

Because the concentrations of inflammation-related proteins in the ELGAN study varied with gestational age, and with the post-natal day of collection^28^, we divided our sample into 15 groups defined by gestational age category (23–24, 25–26, 27 weeks), and post-natal day of blood collection (1, 7, 14, 21, and 28). Because we were interested in the contribution of both high and low concentrations, and the concentrations of most proteins did not follow a normal distribution, the distribution of each protein’s concentration was divided into quartiles among children in each of the 15 groups (three gestational age groups, five collection days).

### Data analyses

We tested three null hypotheses. First, elevated concentrations of each protein (defined as in the top quartile) are not associated with increased ASD risk. Second, among children who do not have ASD, elevated concentrations of each protein are not associated with increased risk of SRS-defined social impairment. Third, proteins with neurotrophic properties, and those with anti-inflammatory properties, do not influence the association between selected inflammation-related proteins and either outcome.

To test our hypotheses, we created three sets of logistic regression models of increased ASD risk and the same three sets of models for increased risk of SRS-defined social impairment among children who did not meet ASD criteria. We began by calculating odds ratios using information from individual day protein measurements (i.e., highest quartile blood concentration for gestational age). Next, we calculated odds ratios based on 2 days of single-protein elevations in the early or late epoch (post-natal days 1, 7, 14, and 21, 28, respectively). Finally, we generated our main findings by calculating odds ratios based on sets of proteins that were elevated on multiple days during the early or late epoch.

We examined two groups of protein sets. First, we paired individual proteins with neurotrophic properties, or individual proteins with anti-inflammatory properties, to an inflammation-related protein whose recurrent elevations are associated with increased risk of ASD or SRS-defined social impairment during the early or late epoch. Second, we paired these same neurotrophic and inflammation-related proteins with TNF-α and (in separate models) IL-8. We chose these two proteins based on evidence that dysregulation of each molecule might provide key information about increased risk of ASD^29–37^, and evidence of association with SRS scores among children who meet ASD criteria^32^. In addition, in the full sample recurrent/sustained top quartile concentrations of TNF-α and IL-8 were strongly associated with increased risk of brain ultrasound abnormalities during the first post-natal weeks^38^, and with attention deficit hyperactivity disorder at age 10 years^39^. Thus, each protein set model involved two proteins (e.g., BDNF (a neurotrophic protein) and SAA (a protein with inflammation-initiating properties)). We compared children whose blood concentrations of just one of these two proteins was elevated on multiple days (i.e., either the neurotrophic protein or the inflammation-related protein concentration was in the top quartile), and those whose concentrations of both of the two proteins was recurrently elevated, to the remaining reference group of children who had neither protein concentration in the top quartile on 2 days during the early or late epoch.

In the ELGAN cohort, the prevalence^20^ and risk profile^1^ of ASD among children with IQ <70 differ from those whose IQ were ≥70. We wanted to examine the relationship between top quartile protein concentrations and ASD separately in both IQ groups, but the relatively small number of children with an IQ <70 who had proteins measured, about half of who met ASD diagnostic criteria (*N* = 24/52), precluded this. Consequently, we examined the relationship between top quartile protein concentrations and ASD, and between top quartile protein concentrations and SRS-defined social impairment (as indicated by an SRS total score ≥65), only among children whose DAS-II verbal or non-verbal reasoning scores were ≥70.

Children in the ELGAN study cohort who had top quartile concentrations of inflammation-related proteins were no more likely than their peers who had lower concentrations to have a mother who had limited educational achievement, a low score on the Kaufman Brief Intelligence Test, Second Edition™ (KBIT-2™), or was eligible for government-provided medical care insurance (Medicaid)^40^. Thus, confounding by social class is minimized, nor were other potential confounders, such as sex, associated with systemic inflammation. Alternatively, low gestational age and fetal growth restriction were associated with elevated protein concentrations^41^, and with ASD^1^, as well as with a high SRS total score ≥65 (unpublished data). Consequently, we adjusted for gestational age category (23–24, 25–26, 27 weeks) and birthweight *Z*-score <−1. Odds ratios (ORs) with 95% confidence intervals (CI) that do not include the null estimate of association (OR “1.0”) are statistically significant. The relevant data underlying this study are subject to ethical/legal restrictions. Interested researchers can visit policies and procedures for information about data access and analysis from the ELGAN study website (http://www.bmc.org/Documents/PAD-Elgan-Study.pdf).

## Results

Of the 794 children whose verbal or non-verbal IQ was ≥70, 763 were evaluated for ASD and had proteins measured in blood on 2 separate days; 36 (5%) met ASD criteria at age 10 years (Supplement Table 1). Of the 720 children whose verbal or non-verbal IQ was ≥70 who did not have ASD, 130 (18%) had a SRS total score ≥65.

Odds ratios estimated by models of increased risk of ASD and (separately) SRS-defined social impairment that we created using information from individual proteins repeatedly elevated during the early or late epoch are, respectively, displayed side by side in Supplement Figures S1 and S2. We generated our main findings using information from protein sets that were repeatedly elevated during the early or late epoch (Supplement Figures S3–S5). To acknowledge that statistical power was limited, and yet not discard information from non-significant odds ratios, we used different symbols in the figures to identify statistically non-significant odds ratios that were ≥2.0.

We prepared Table 1 to summarize in what ways high concentrations of proteins with neurotrophic properties (i.e., EPO, BDNF, IGF-1, VEGF, VEGF-R2, PIGF, Ang-1, Ang-2) and proteins with anti-inflammatory properties (e.g., IL-6R, MMP-9, RANTES) appear to modulate the risk of ASD or (separately) SRS-defined social impairment that was associated with high concentrations of selected pro-inflammatory proteins (e.g., SAA, IL-6, TNF-α, or IL-8) occurring on multiple days of the early or late epoch.

**Table 1:**
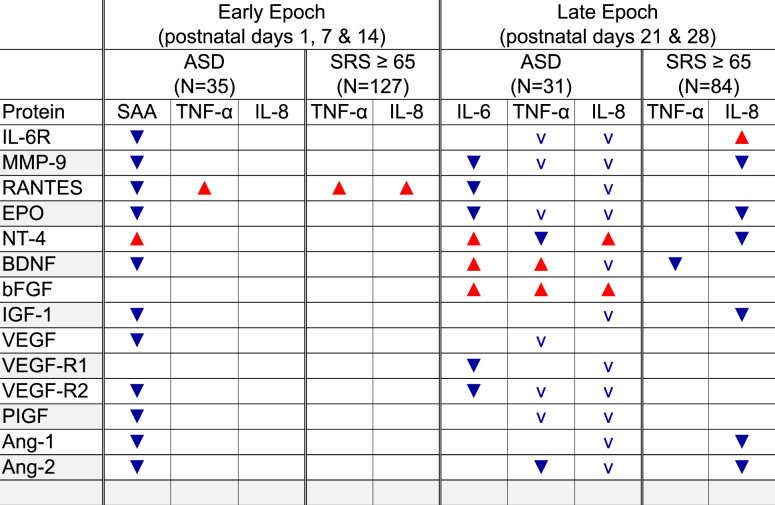
This is a summary of the main findings displayed in Supplement Figures S3–S5

indicates increased risk of the entity at the top of sets of columns when the concentrations of both the pro-inflammatory protein listed in each column heading and the protein listed on the left are high, while  indicates the increased risk associated with the protein in the column heading is reduced in the presence of a high concentration of the protein on the left. The small  in the late-epoch column for ASD indicates a non-significant ASD odds ratio of 2.0 or more associated with high concentrations of IL-8, but only when the concentration of the protein on the left is not high.

### Risk of ASD associated with sets of proteins measured in blood during the early and late epochs

Because increased risk of ASD was associated with a high concentration of SAA on 2 days during the early epoch (OR (95% CI): 2.5 (1.2, 5.3)), and with top quartile IL-6 on both days of the late epoch (OR (95% CI): 2.6 (1.03–6.4)) (Supplement Figure S2), we wanted to see if a high concentration of a neurotrophic protein, or of a protein with anti-inflammatory properties on 2 days during the early or late epoch modulated these associations (Summary Table 1 and Supplement Figure S 3). The elevated risk of ASD associated with a recurrent top quartile concentration of SAA in the early epoch, and with a top quartile concentration of IL-6 on both days of the late epoch, is prominently modulated by both neurotrophic and anti-inflammatory proteins (i.e., the odds ratios in Supplement Figure S3, column 3 differ from those in columns 1 and 2).

When the concentration of a neurotrophic protein, or a protein with anti-inflammatory properties, was not in the top quartile during the early epoch, a recurrent top quartile concentration of SAA was associated with two-to-fourfold increased risk of ASD in 12 of the 14 models (OR (95% CI) range: 2.3 (1.05–5.2) to 3.8 (1.7–8.8); Supplement Figure S3, column 3). By contrast, when the neurotrophic protein, or a protein with anti-inflammatory properties, was in the top quartile, recurrent elevation of SAA is associated with increased risk of ASD in only one of the 14 models, when NT-4 was also elevated (OR (95% CI): 4.6 (1.1–18); Supplement Figure S3, column 1). A similar, but somewhat less impressive, pattern of association is seen during the late epoch in models with top quartile concentrations of IL-6 and each of these proteins. When the concentration of a neurotrophic protein, or a protein with anti-inflammatory properties, was not in the top quartile, a recurrent top quartile concentration of IL-6 was associated with two-to-threefold increased risk of ASD in 7 of the 14 models (OR (95%CI) range: 2.5 (1.6–17) to 3.1 (1.2–8.1)).

By contrast, when the neurotrophic protein, or a protein with anti-inflammatory properties, was not in the top quartile, a recurrent top quartile concentration of IL-6 during the late epoch is associated with increased ASD risk in only 3 of the 14 models, when combined with elevated concentrations of BDNF (OR (95% CI): 7.8 (1.3–48)), bFGF (OR (95% CI): 12 (1.7–84)) or NT-4 (OR (95%CI): 37 (2–694)). Indeed, the increased risk of ASD associated with a top quartile NT-4 concentration was seen even when the late-epoch concentration of IL-6 was not in the top quartile (OR (95% CI): 3.6 (1.4–9)).

### Risk of ASD and (separately) of SRS-defined social impairment associated with sets of proteins involving TNF-α or IL-8 measured in blood during the early and late epochs

Based on a priori evidence of association between ASD and elevated blood concentrations of specific proteins, and also because top quartile TNF-α and IL-8 concentrations were associated with increased risk of ASD and/or a high SRS total score on post-natal days 14, 21, and 28 (Supplement Figure S1) in our sample, we wanted to see if an association between either outcome and recurrent elevation of each protein during the early or late epoch was modulated by an elevated concentration of a neurotrophic protein or a protein with anti-inflammatory properties (Summary Table 1, Supplement Figures S4 and S5). Increased ASD risk did not occur with high concentrations of either TNF-α or IL-8 on 2 days in the early epoch, not even when the concentrations of the neurotrophic proteins were below the 75th percentile (Supplement Figure S4). Only the combination of recurrent top quartile RANTES and elevated IL-8 concentration was associated with increased risk of high SRS total score during the early epoch (OR (95% CI): 2.8 (1.3–6)); none of the other 13 models identified protein sets whose recurrent elevations were associated with social impairment during the early epoch.

By contrast, during the late epoch, when the concentrations of NT-4 and Ang-2 were not recurrently elevated, top quartile concentrations of TNF-α on both days were associated with increased ASD risk (OR (95% CI): 3.2 (1.1–9) and 2.8 (1.1–7.7), respectively). When the neurotrophic protein or a protein with anti-inflammatory properties was not in the top quartile, recurrent elevation of TNF-α was non-significantly associated with twofold or more increased risk of ASD in 9 of the 12 remaining models. (Supplement Figure S5, column 3). A similar pattern of association occurred during the late epoch in models including recurrent elevations of IL-8. When the neurotrophic protein or a protein with anti-inflammatory properties was not in the top quartile, recurrent elevation of IL-8 was non-significantly associated with twofold or more increased risk of ASD in 11 of 14 models. Increased ASD risk also occurred with top quartile concentrations of both proteins on both days of the late epoch for combinations of TNF-α with BDNF (OR (95% CI): 5.6 (1.4–23)) and bFGF (OR (95% CI): 6.1 (1.1–35)), and also for combinations of IL-8 with NT-4 (OR (95%CI): 18 (2.4–130)) and bFGF (OR (95%CI): 12 (1.6–81)). A recurrent top quartile NT-4 concentration was associated with increased ASD risk even when combined with a low TNF-α concentration during the late epoch.

The most prominent pattern of association with statistically significant elevations of SRS-defined social-impairment risk occurred during the late epoch with a top quartile pro-inflammation protein concentration in combination with a low neurotrophic (or anti-inflammatory) protein concentration on both days; this pattern occurred with combinations of TNF-α and IL-6R, RANTES, BDNF, PIGF, and Ang-1 (OR (95% CI) range: 1.9 (1.01–3.6) to 2.3 (1.2–4.3)), and with combinations of IL-8 and EPO, NT-4, IGF-1, Ang-1, and Ang-2 (OR (95% CI) range: 2 (1.05–4) to 2.1 (1.1–4.1)). Statistically significant elevations in risk of a total SRS ≥65 also occurred with a combination of top quartile IL-8 and top quartile IL-6R, concentrations on both days of the late epoch (OR (95% CI): 4.4 (1.2–16)). In addition, increased risk of SRS-defined social impairment occurred when the concentration of TNF-α was below the top quartile and the concentration of BDNF was not (OR (95% CI): 2.1 (1.05–4.2)).

## Discussion

We have three main findings. First, after excluding children whose verbal or non-verbal IQ was ≥70, those who developed SRS-defined social limitations, and children diagnosed with ASD at age 10 years, have newborn blood protein profiles that differed from peers who developed neither of these two conditions. Second, top quartile concentrations of neurotrophic proteins, and proteins with anti-inflammatory properties, do, indeed, appear to moderate the increased risk of ASD associated with repeated top quartile concentrations of an inflammation-related protein during the first post-natal month. Third, neurotrophic proteins seem to modulate the increased risk of a high SRS total score associated with a top quartile concentration of an inflammation-inducing protein on post-natal days 21 and 28, but not before, in contrast to the models of increased ASD risk.

### Neurodevelopmental outcomes of children born very preterm

We do not know why children born very preterm are at increased risk of neurodevelopmental disorders including ASD and its’ correlates; vulnerability of brain maturation processes^42^, a paucity of neuroprotective factors^39,43^, post-natal physiologic instability^42,44^, neonatal illnesses (e.g., bacteremia^45^), and systemic inflammation-related phenomena appear to contribute to the increased risks^8,46^. In the ELGAN study cohort, severe fetal growth restriction is the most strongly associated antecedent of ASD without intellectual disability (OR (95% CI): 9.9 (3.3–30))^1^. In agreement with others, we have also found that extremely low gestational age at delivery is associated with a several-fold increased risk of ASD irrespective of intellectual ability^47^. Because ASD and social impairment have been associated with sonographic images of white matter irregularities (ventriculomegaly and disrupted microstructure integrity/connectivity)^10,13,48–61^, and because white matter injury (WMI) among children born very preterm are associated with systemic inflammation-related phenomena^8,46,62,63^, our findings might reflect these relationships.

### Inflammation-related proteins, neurotrophins, and neurodevelopmental disorders

Under an allostasis framework, “…acute stress enhances immune function whereas chronic stress suppresses it, [which] can be beneficial for some types of immune responses and deleterious for others”^64^. From this perspective, the mechanisms that alter brain structure or function might reflect the consequences of enhancing inflammation resolution^65^, or enhancing repair^66^, or most likely a mix of both^67–69^. Neurotrophins and angiotrophins are likely to participate in such processes.

Though the relationships among neurotrophins and inflammation are complex, prior studies have provided evidence of anti-inflammatory attributes^70,71^, which might confer “neuroprotection” or otherwise influence “neurovulnerability.” Indeed, “..the immune system and the nervous system are anatomically connected, mechanistically communicate and reciprocally influence the other’s function”^72^, there is evidence of co-regulation of inflammation and social behavior^73^, and alterations in serum level of specific growth factors have been implicated in the etiology, symptoms, and progression of some psychiatric disorders, including ASD, cognitive, mood and social disabilities^16–18,74^.

In studies of our cohort, elevated concentrations of inflammation-related proteins on only 1 day are less likely to convey information about the risk of developmental disabilities than are elevated concentrations on multiple days^8^. We have also found that elevated concentrations of an inflammation-related protein on multiple days are best evaluated in the presence and absence of elevated concentrations of neurotrophic proteins^39^.

The increased risk of ASD in the presence of high concentrations of both an inflammatory and a neurotrophic protein might be an indicator of the release of both proteins into the circulation. A likely inference then is that the high concentrations do not contribute to the onset of brain damage or dysfunction, but rather are a tertiary consequence of processes already initiated. Evidence from animal models supports this possibility^63^.

Another possibility is that a high concentration of one protein contributes to processes which then result in the release of the other protein or the release of signals to increase the synthesis of the other protein. Although the etiologic significance of these differences remains to be identified, inflammation seems involved in the pathogenesis of both ASD and SRS-defined social limitations.

### Findings from other studies

Prior studies have also demonstrated underexpression of VEGF^32^, TGF-β1^30^, NT-3^37^, NT-4^75,76^, HGF^77^, EGF^77–79^, and IP-10^80^ in blood obtained from children with a current or later diagnosis of autism. Affected children are also more likely than others to have high blood concentrations (or messenger RNA expression) of some neurotrophic proteins (e.g., BDNF^17,81,82^ and NT^83^) and such inflammation-related proteins as IL-1b^32,36,84,85^, IL-1RA^35^, IL-4^85^, IL-5^35^, IL-6^36,84^, IL-8^34^, IL-12 (p40)^36^, IL-12 (p70)^35^, IL-13^35^, IL-17^84^, GRO-α^35^, TNF-α^29,86^, RANTES^80^, MIP-1-α^80^, MCP-1^87^, MIP-1α^80^, and MIP-1β^80^.

### Molecular risk profiles

ASD is currently diagnosed entirely according to behavioral criteria, although efforts are underway to identify concurrent biological markers for disease risk and early diagnosis^88^. We, however, have studied biomarkers of very early post-natal exposures and characteristics to better understand why children born extremely preterm are at increased risk of ASD and social-impairment limitations. We do not encourage classifying children’s risks based on concurrent concentrations of the proteins we measured.

### Limitations and strengths

Among the strengths of this study are the large sample size, enrollment based on gestational age and not birthweight, modest attrition, protein measurements of high-quality^89^ and high-content validity^90^ longitudinal measurements of blood protein concentrations rather than single “snap shots”^91^, and high-quality assessments of ASD and social limitations 10 years later^92^. To avoid type II errors whose likelihood is increased with inappropriate adjustment for multiple comparisons^93^, we did not perform such adjustments. This might have increased the risk of type I errors. Despite the large size of the ELGAN study cohort, we had limited power, but nevertheless were able to identify statistically significant associations in this sample. Our findings are generalizable to children born extremely preterm whose verbal or non-verbal IQ was ≥70; even though we excluded children whose verbal and non-verbal IQ were <70, it is possible that some of the remaining intellectual deficits influenced our findings.

## Conclusion

High concentrations of neurotrophins in newborn blood appear to modulate the increased risks of ASD associated with systemic inflammation during the first post-natal month; a similar pattern of association was observed for SRS-defined social, but only on post-natal days 21 and 28.

## Electronic supplementary material

Supplemental Results

Supplement Figure 1

Supplement Figure 2

Supplement Figure 3

Supplement Figure 4

Supplement Figure 5
