# Supplementary material for: Elevated protein concentrations in newborn blood and the risks of autism spectrum disorder, and of social impairment, at age 10 years among infants born before the 28th week of gestation
Source: Transl Psychiatry. 2018 Jun 8;8:115. doi: 10.1038/s41398-018-0156-0 (PMC5993745; doi:10.1038/s41398-018-0156-0)
Supplement: Supplementary file 1 — Supplemental Results [file 41398_2018_156_MOESM1_ESM.docx]

**SUPPLEMENT MATERIALS**

**Results**

The number of children in the ASD-assessed sample for whom blood concentration measurements were available declined from day 7 (N = 729) to day 14 (N = 656) to day 21 (N = 602) to day 28 (N = 565). Among the ASD-assessed sample, 736 children provided two or three specimens of blood during the first two postnatal weeks, while only 490 provided two specimens from the following two weeks. Among the SRS-assessed sample, 695 children provided two or three specimens of blood during the first two postnatal weeks, and 455 provided two specimens from the following two weeks.

ASD risks based on individual day measurements (Supplement Figure S1)

On days 1, and 7, the top quartile concentration of not a single protein was associated with increased or decreased risk of ASD. On day 14, the top quartile concentration of SAA was associated with increased risk (odds ratio [OR] = 2.5; 95% confidence interval

[CI]: 1.1, 5.5), while a top quartile concentration of MMP-9 was associated with decreased risk (OR = 0.2; 95% CI: 0.05, 0.8). On day 21, a top quartile concentration of NT-4 was associated with increased risk (OR = 2.1; 95% CI: 1.01, 4.3), as was a top quartile concentration of TNF-α on day 28 (OR = 2.5; 95% CI: 1.2, 5.2).

Increased risks of a high total SRS score were associated with top quartile concentrations of TNF-R2 and TSH on day 1, TSH on day 7, IL-8, ICAM-1, IGFBP-1, and VEGF-R2 on day 21, and IL-8, ICAM-1 on day 28. The risks of a high score were reduced among children who had top quartile concentrations of NT-4 on day 14, and bFGF and VEGF-R1 on day 28.

ASD risks based on two days of elevation in the early and late epochs (Supplement Figure S2)

A top quartile concentration of SAA on two separate occasions a week apart during the first two weeks was associated with increased risk of ASD (OR = 2.5; 95% CI: 1.2, 5.3), while a top quartile concentration of IL-6 on both specimens collected during the third and fourth postnatal weeks was also associated with increased ASD risk (OR = 2.6; 95% CI: 1.03, 6.4). During the early epoch, a statistically-significant elevated risk of a total SRS ≥ 65 occurred only with a top quartile concentration of IL-8 combined with a top quartile concentration of RANTES.

Increased risk of a total SRS ≥ 65 was associated with elevated concentrations of TSH on two of the three early epoch days (*i.e.,* days 1, 7, and 14), while top quartile concentrations of no protein on both days of the late epoch (days 21 and 28) was associated with increased risk. Elevated concentrations of none of the 27 proteins were associated with reduced risk on two of the three early epoch days or on both of the late epoch days.

**Tables**

Table S1. Sample description

|  | Yes | No |
| --- | --- | --- |
| Enrolled | 1506 |  |
| Survived to 10 years | 1198 | 308 |
| Returned for an DAS assessment at age 10 years | 874 |  |
| Had a DAS verbal ≥ 70 OR DAS nonverbal ≥ 70 | 794 | 81 |
| Had Proteins measured in blood collected on ≥ 1 day | 783 | 11 |
| Had Proteins measured in blood collected on ≥ 2 days | 764 | 30 |
| Child evaluated for ASD | 763 | 1 |
| ASD diagnosis | *36* | *727* |
| ASD no and SRS total evaluated | 720 |  |
| SRS ≥ 65 | *130* | *590* |
