# Supplementary figures and images for: Elevated protein concentrations in newborn blood and the risks of autism spectrum disorder, and of social impairment, at age 10 years among infants born before the 28th week of gestation
Source: Transl Psychiatry. 2018 Jun 8;8:115. doi: 10.1038/s41398-018-0156-0 (PMC5993745; doi:10.1038/s41398-018-0156-0)

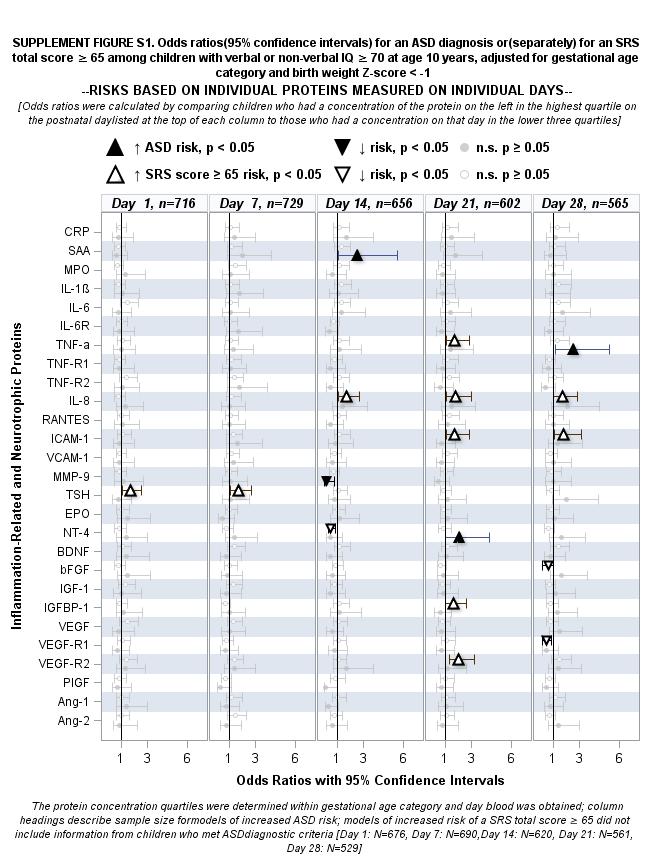

Supplement: Supplementary file 2 — Supplement Figure 1 [file 41398_2018_156_MOESM2_ESM.jpg]

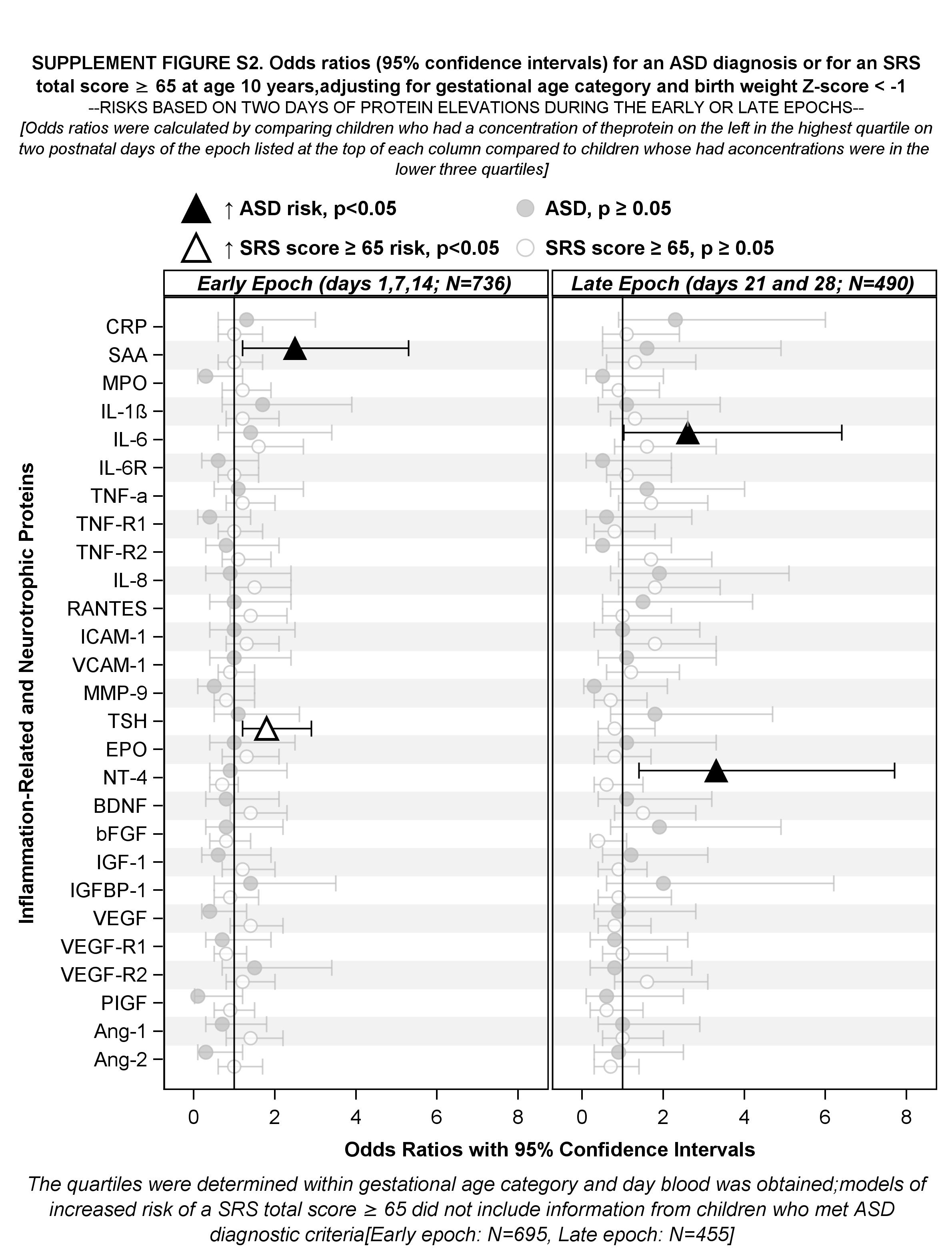

Supplement: Supplementary file 3 — Supplement Figure 2 [file 41398_2018_156_MOESM3_ESM.jpg]

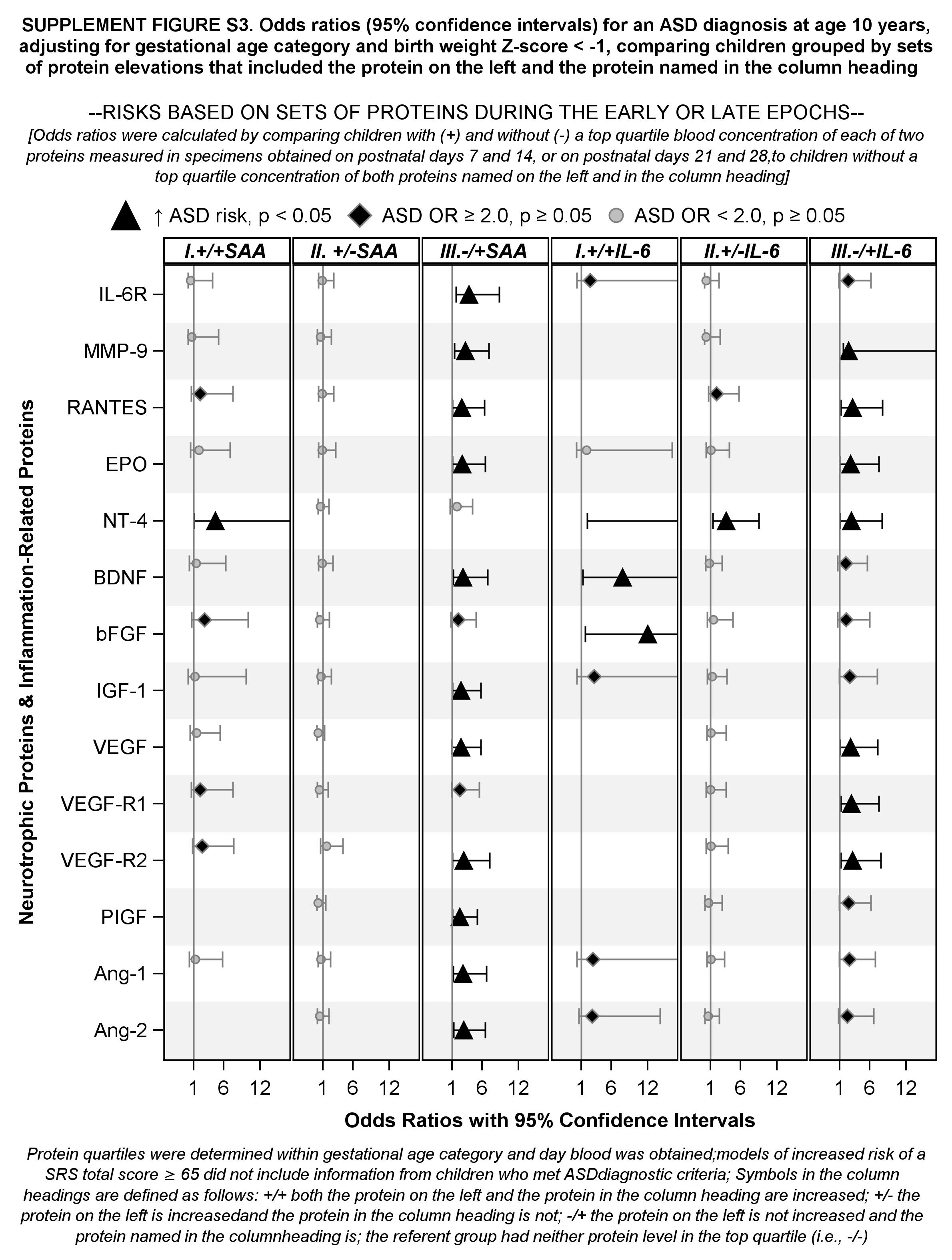

Supplement: Supplementary file 4 — Supplement Figure 3 [file 41398_2018_156_MOESM4_ESM.jpg]

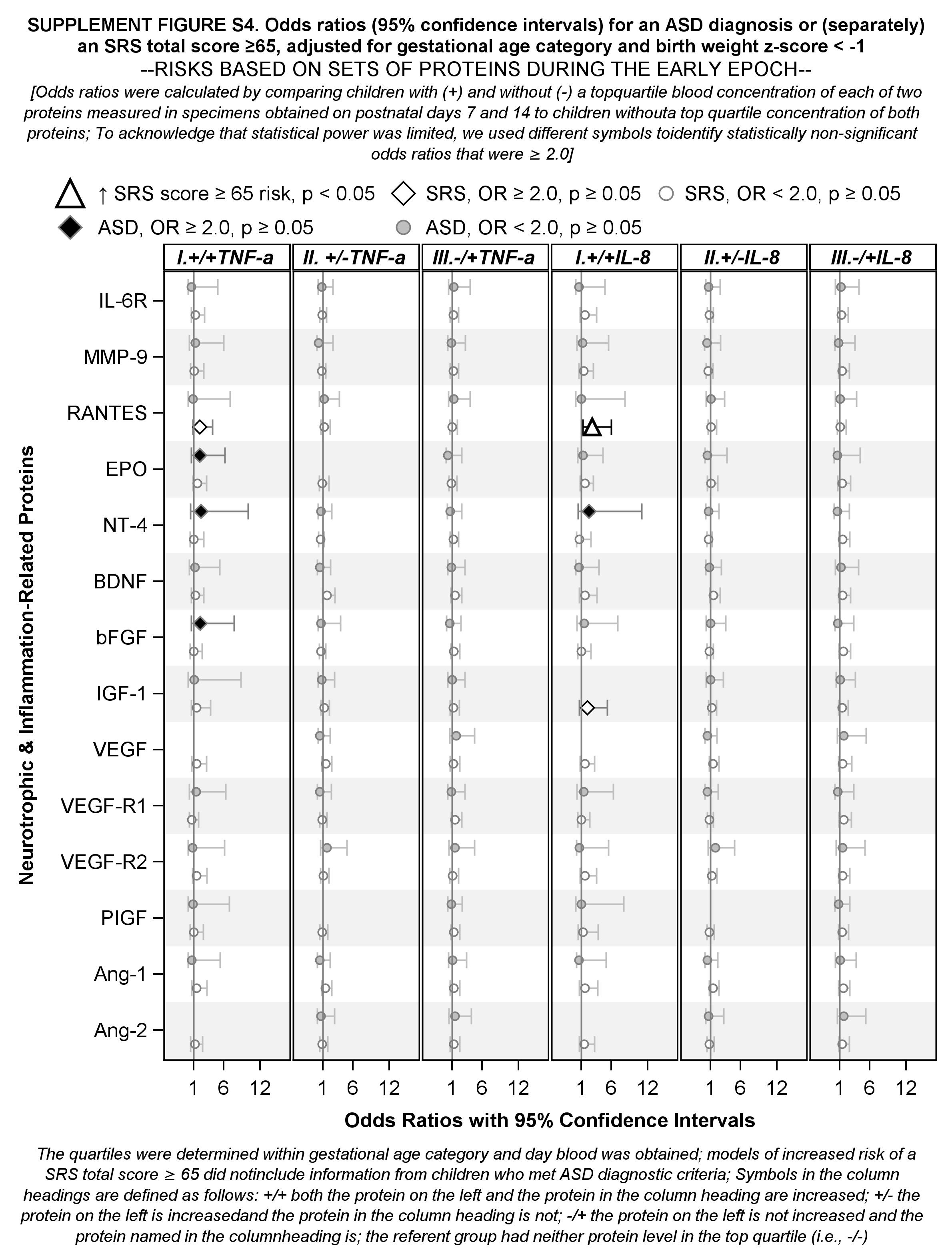

Supplement: Supplementary file 5 — Supplement Figure 4 [file 41398_2018_156_MOESM5_ESM.jpg]

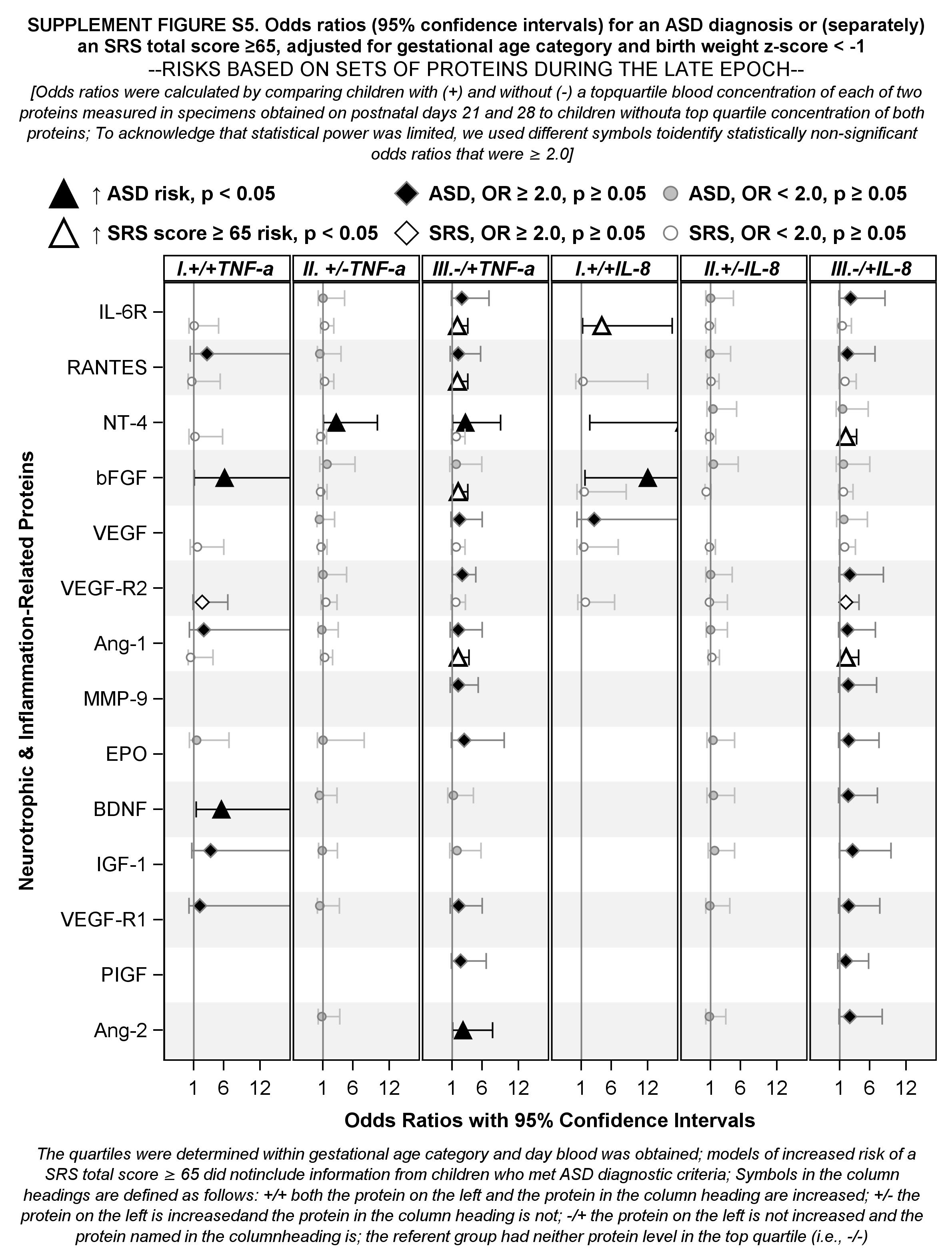

Supplement: Supplementary file 6 — Supplement Figure 5 [file 41398_2018_156_MOESM6_ESM.jpg]
